# Guillain-Barré syndrome and severe infection following chemotherapy for peripheral T-cell lymphoma: A case report

**DOI:** 10.3892/ol.2014.2541

**Published:** 2014-09-16

**Authors:** YANG-YANG MA, LEI ZHANG, DA-LIANG ZHANG, WEN-SHUO LIU

**Affiliations:** Department of Oncology, The First Affiliated Hospital of Zhengzhou University, Zhengzhou, Henan 450002, P.R. China

**Keywords:** peripheral T-cell lymphoma, Guillain-Barré syndrome, immune reconstruction

## Abstract

Guillain-Barré syndrome (GBS) is a rare complication of malignant lymphoma. The current study describes a case of GBS in a patient with peripheral T-cell lymphoma not otherwise specified (PTCL-NOS). A 47-year-old male was admitted to the First Affiliated Hospital of Zhengzhou University (Zhengzhou, China) with systemic multiple subcutaneous nodules and was diagnosed with stage IV high-grade PTCL-NOS (according to the Revised European American Lymphoma Classification). During chemotherapy, severe infection and progressive flaccid quadriparesis appeared, which eventually developed to respiratory muscles paralysis. The clinical course and neurological examination were consistent with GBS. Following mechanical ventilation and intravenous immunoglobulin administration, the neurological symptoms were in remission after one month. Three months later, the patient achieved complete remission without any treatment during this period. We hypothesized that immune reconstruction may have a significant role in this phenomenon.

## Introduction

Peripheral T-cell lymphoma (PTCL) is a rare and highly aggressive malignancy with a poor outcome. The condition accounts for ~12% of all lymphoid malignances and is roughly divided into specified and not otherwise specified forms (1). The not otherwise specified forms, forming 60–70% of T-cell lymphomas, cannot be further classified according to morphology, phenotype or conventional molecule biology (2). Immunohistochemistry generally detects T-cell associated molecular expression and clonal rearrangements of T-cell receptor (TCR) encoding genes. International T-cell lymphoma data has shown that the overall survival (OS) rate of PTCL-NOS at 10 to 15 years was 10% (3). Treatment advances in PTCLs have been slow compared with other lymphomas. The effect of conventional chemotherapy is poor, with rates of relapse-free survival for 5 years recorded as <30% (4). Cyclophosphamide, doxorubicin, vincristine and prednisone (CHOP) or a CHOP-like regimen combined with anthracyclines forms the first-line therapy for PTCL. Gemcitabine has proven to be effective in pretreated PTCL patients, even in the long term, thereby providing rationale for providing it as a frontline therapy (5). A multicenter study demonstrated promising results from the response of pegylated liposomal doxorubicin monotherapy, with a low rate of severe adverse reaction found in patients with primary cutaneous T-cell lymphomas when the treatment was compared with other chemotherapy protocols (6). It is necessary to improve the outcome for PTCL patients by utilizing novel therapeutic strategies and the incorporation of novel drugs into these therapeutic regimens.

Guillain-Barré syndrome (GBS) is an acute inflammatory polyneuropathy characterized by acute areflexic paralysis and rapidly progressive impairment, predominantly of motor function (7). GBS is widely held to be an immunologically-mediated disease consisting of at least four subtypes of acute peripheral neuropathy, including acute inflammatory demyelinating polyneuropathies, acute motor axonal neuropathy, acute motor-sensory axonal neuropathy, Miller Fisher syndrome and acute sensory neuropathy (8). The majority of cases are caused by the immune response following a preceding infection, such as that of *Campylobacter jejuni*, *Cytomegalovirus*, Epstein-Barr virus or varicella-zoster virus (7–9). Albuminocytological dissociation (high levels of protein and normal cell counts) can be found in the cerebrospinal fluid (CSF). GBS presents with a self-limiting course and several controlled clinical trials have shown that plasma exchanges and intravenous immunoglobulin shorten the time to recovery when used in the early stages of the neuropathy (10). Chemotherapy-treated patients with lymphoma frequently develop neurological abnormalities, including infiltration of the nervous system by the lymphoma and drug toxicity. Nevertheless, a diagnosis of GBS should be considered. The present study reports the occurrence of GBS in a patient with PTCL-NOS. Written informed consent was obtained from the patient.

## Case report

A 47-year-old male was admitted to the First Affiliated Hospital of Zhengzhou University (Zhengzhou, Henan, China) with multiple systemic subcutaneous nodules and a left testicular neoplasm. The patient had a performance status score of 1 according to the Eastern Cooperative Oncology Group classification. Cardiopulmonary auscultation revealed normal sounds and cervical, axillary and inguinal lymphadenopathy was palpable, however, the liver and spleen were not palpable. The neurological examination was unremarkable and the laboratory tests results at admission were as follows: A white blood cell count of 4.9×10^9^/1 (normal range, 4.0–10.0×10^9^/l) without atypical lymphocytes, a hemoglobin concentration of 140 g/dl (normal range, 120–160 g/l), a thrombocyte count of 381×10^9^/1 (normal range, 100–300×10^9^/l) and a lactate dehydrogenase level of 177 U/l (normal range, 135–215 U/l). Bone marrow aspirate examination showed no obvious abnormalities, while the histopathology of the nodules on the left side of the patients back revealed PTCL-NOS according to the REAL classification (?). The immunohistochemistry was positive for T-cell markers, including cluster of differentiation (CD)3, CD4, CD43, CD5 and TCRα/β, with a Ki-67 of 30–40%, but negative for TCRγ/δ. The thoracic and abdominal computed tomography (CT) revealed bilateral pulmonary and renal nodules, nodules on the spleen and a few enlarged hilar lymph nodes. In addition, the nasopharyngeal CT showed mucosal thickening within the nasal cavity and turbinate, as well as multiple lymphadenopathy around the parotid region and carotid artery area. According to the Ann-Arbor classification, the patient had stage IV high-grade PTCL-NOS (11). As a result, treatment with two cycles of gemcitabine (800 mg/m^2^, days 1 and 8), cisplatin (20 mg/m^2^, days 1–4), prednisone (60 mg/m^2^, days 1–5) and thalidomide (200 mg/m^2^, days 1–21) every 21 days, was initiated immediately. Owing to the occurrence of new subcutaneous nodules, the dosage regimen was adjusted to a treatment with gemcitabine (800 mg/m^2^, days 1 and 8), pegylated liposomal doxorubicin (20 mg/m^2^, day 1), pegaspargase (2,500 IU/m^2^, day 1), dexamethasone (15 mg/m^2^, days 1–5) and thalidomide (200 mg/m^2^, days 1–21) every 21 days for two cycles. The patient achieved partial remission following four cycles of treatment.

Subsequently, the patient complained of coughing accompanied by excessive phlegm, chest congestion, loss of appetite and fatigue. The thoracic CT scan showed bilateral pulmonary fungal infection, bacterial infection, interstitial involvement and bilateral pleural effusion. Treatments with antifungal agents (voriconazole; 6 mg/kg every 12 h for 1 day, then 4 mg/kg every 12 h for three weeks ), antibacterial drugs (500 mg imipenem every 8 h and 200 mg levofloxacin every 12 h for two weeks), granulocyte colony-stimulating factor and methylprednisolone were commenced and the patient’s symptoms and signs improved markedly.

Following these treatments, symmetric weakness of the lower extremities with paresthesia ensued, with subsequent progression to the upper limbs. Difficulty in swallowing and tongue tip numbness were reported a few days later. A neurological examination revealed no cognitive deficits and the cerebellar function was normal. Cranial nerve examination showed moderate bilateral facial diplegia and motor examination revealed a symmetrical flaccid quadriparesis (strength, 2/5 to 3/5, according to Medical Research Council muscle strength grades) (12). Sensory examination demonstrated a mild decrease in the ability to sense vibration in the feet, as well as reduced sensation in all fingers and toes bilaterally. In addition, reflexes were absent in all limbs. The cerebral and spinal cord magnetic resonance imaging (MRI) revealed no clear abnormalities. The acute progression was similar to that of GBS and therefore, the CSF was examined; increased protein and normal mononuclear cells were found. Electroneurography revealed a reduction in the motor conduction velocity and motor amplitude. In addition, electromyography of the left anterior tibialis and left vastus lateralis muscles showed no signs of denervation. According to the clinical course and neurological tests, a diagnosis of GBS was determined. Treatment was initiated with intravenous human immunoglobulin (440 mg/m^2^/day) for five consecutive days. The quadriparesis did not stabilize and respiratory muscle paralysis developed, which eventually led to respiratory failure. Mechanical ventilation was instituted immediately for three weeks followed by an additional course of intravenous human immunoglobulin (400 mg/kg/day for five days). The patient’s respiratory function recovered and the progress of the paresis was halted a month later. The patient’s exercise capacity and sensibility recovered in significant measures.

Three months later, the patient was discharged for later re-examination. During this period, the patient did not accept any treatments for lymphoma. The multiple systemic subcutaneous nodules and left testicular neoplasm disappeared, and CT of the chest and whole-body diffusion-weighted MRI showed complete response of the lymphoma. Six months later, the positron emission tomography-CT showed no residual tumor activity. No further chemotherapy was administered and to date, no evidence of recurrence has been observed.

## Discussion

Neurological symptoms are common in patients with malignant lymphoma who are undergoing treatment. The differential diagnosis mainly incorporates nervous system invasion together with lymphoma and drug neurotoxicity, all well-known characteristics. Progressive symmetrical paralysis of the limbs with areflexia led to the suspicion of GBS in the present patient. Subsequent albuminocytological dissociation of the CSF and electroneurography confirmed the diagnosis of GBS. GBS has been described in association with a number of different hematological neoplasms, but particularly with Hodgkin’s lymphoma (HL) and during the post-transplantation period (13–17). However, the appearance of GBS in association with non-HL (NHL) is extremely rare and few cases have been described in the literature (18–21). Plasma exchange and intravenous immunoglobulin play a role in hastening the recovery of patients with GBS. The combination of plasma exchange and a course of intravenous immunoglobulin is not prominently better than using plasma exchange or intravenous immunoglobulin alone (10). The present patient, with PTCL-NOS complicated by severe infection and GBS, achieved complete remission following efficient anti-infective therapy and immunotherapy. This report is the first to explore the role of immune reconstruction in malignant lymphoma with GBS.

The human immune system is a complicated network of organs, glands and tissues, which protects the body from foreign substances, allowing for control of the response to pathogens and enabling self-tolerance (22,23). In our natural defenses against carcinogenesis, the immune system defense mechanisms are extremely effective and act as a final barrier (24). It is common knowledge that certain factors can cause deregulation of the checks and balances in the body, eventually increasing the risk of an uncontrolled response and proliferation, which presents as lymphoma or lymph proliferative disorders. Abundant innate immune cells (macrophages, mast cells, neutrophils and T-regulatory cells) infiltrate the body in NHL. These cells are correlated with an increased risk of immune evasion, neoangiogenesis and a poor prognosis. Not only neoplastic cells, but also specific immune cells polarized towards a Th2 phenotype in order to evade antitumor immunity are the most relevant mechanisms of immune escape (25). Autoimmune diseases form another heterogeneous group that lack self-tolerance and lead to chronic inflammatory responses, and as with most complex conditions, lymphoma genesis likely involves interaction at the genetic and environmental levels. Therefore, autoimmune diseases and lymphoma share the multistep-process that eliminates checkpoints which prevent the clonal proliferation of uncontrolled cells (25).

Autoimmunity and immune-mediated diseases are caused by the failure or break down of immunological tolerance; the immunocompromised state can be due to a transient factor, such as infection with a pathogen capable of controlling the immune response. We suggest a comprehensive hypothesis about the pathogenesis of GBS that is based on the condition being due to a transient immune deficiency and the fact that, in the majority of cases, GBS follows an infection (26). Such infections may result in the breakdown of immune tolerance, subsequently leading to induction of an immune assault on the peripheral nerves. An increase in the number of immune-mediated attacks may be triggered either by the same infective agents or by secondary infection. We believe that the cellular and humoral immune system conduce to the pathogenesis of GBS. Furthermore, activation of complement has been associated with the initiation of myelin damage, and cytotoxicity also play a vital part in GBS (26).

In the present patient, the effect of the drug could be excluded, since no further treatments were administered following the healing of the severe infection and GBS. In addition, the change of immunological indexes also indirectly demonstrated the association with immunity. Immune reconstruction refers to those immunocompromised individuals using a self-regulating mechanism or adoptive transfer treatment to establish and improve the effective process of the immune system. A reconstructed immune system can regain the ability to attack a neoplasm. Several mechanisms based on immune escape have been proposed, such as the production of immunosuppressive cytokines, the downregulation of human leukocyte antigen on the tumor cell surface and the expression of lymphotoxic molecules by tumor cells. These methods may be interrupted by immune reconstitution, leading to tumor cells apoptosis (27). In addition, the immune microenvironment and cytokine secretion also contribute to this course. In fact, the high complexity of the immune network makes it difficult to clarify the mechanism in this phenomenon underlying tumor-host immune system interactions. Following years of cancer research, success is finally being achieved by strengthening the body’s own defenses to destroy tumors (28). Due to an improved understanding of the fundamental principles of immunology and tumor biology, the immunotherapy of cancer has made significant progress in the past few years. The application of immunotherapy includes the use of antibodies, cytokines, vaccines and cellular therapies (22,27). The further understanding and identification of the impact of immunotherapy on different tumor types, and the development of future multimodal therapy for cancer, combining immunotherapy with chemotherapeutic drugs and molecularly targeted agents, will continue, and eventually lead to benefits for cancer patients from immunotherapy.

In summary, the present study reported a rare case of PTCL-NOS with severe infection and progressive flaccid quadriparesis following chemotherapy, which was subsequently diagnosed as GBS. The patient achieved complete remission following effective anti-infective therapy and immunotherapy. No further chemotherapy was administered and there has been no evidence of recurrence. Immune reconstruction appears to have been the mechanism responsible for this success, however, further evaluation of this mechanism is required and the patient will require follow-up examinations.
